# Trochleoplasty Utilization in the Management of Patellofemoral Instability: Results From an International Survey of Surgeons

**DOI:** 10.1177/23259671241303147

**Published:** 2025-01-13

**Authors:** Brendan A. Williams, Morgan G. Batley, John A. Schlechter, Lauren H. Redler, Moshe Yaniv, Nicole A. Friel, Shital N. Parikh, J. Lee Pace, Beth E. Shubin Stein, Sean Waldron, Stephanie L. Logterman, Kevin Shea, Kendall E. Bradley, Eileen A. Crawford, Elliot Greenberg, Joseph Hannon, Alicia Kerrigan, Megan H.M. Kuba, Jeffrey Albaugh

**Affiliations:** Children’s Hospital of Philadelphia, Philadelphia, Pennsylvania, USA; Perelman School of Medicine at the University of Pennsylvania, Philadelphia, Pennsylvania, USA; Children’s Hospital of Philadelphia, Philadelphia, Pennsylvania, USA; Children’s Hospital of Orange County, Orange, California, USA; Columbia University Medical Center, New York, New York, USA; Dana-Dwek Children’s Hospital–Tel Aviv Sourasky Medical Center, Tel Aviv, Israel; Shriners Hospital for Children Northern California, Sacramento, California, USA; Cincinnati Children’s Hospital Medical Center, Cincinnati, Ohio, USA; Children’s Health Andrew’s Institute, Plano, Texas, USA; Hospital for Special Surgery, New York, New York, USA; Shriner’s Children’s Hospital, Philadelphia, Pennsylvania, USA; Arnold Palmer Hospital for Children, Orlando, Florida, USA; Stanford Hospital, Stanford, California, USA; Duke University, Durham, North Carolina, USA; University of Michigan, Ann Arbor, Michigan, USA; Children’s Hospital of Philadelphia, Philadelphia, Pennsylvania, USA; St Louis Children’s Hospital, St Louis, Missouri, USA; Children’s Hospital of Eastern Ontario, Ontario, Canada; Children’s Orthopaedics of Hawaii, Honolulu, Hawaii, USA; Children’s Hospital of Philadelphia, Philadelphia, Pennsylvania, USA; Investigation performed at the Children's Hospital of Philadelphia, Philadelphia, Pennsylvania, USA

**Keywords:** patellofemoral instability, trochleoplasty, osteotomy, lateral patellar dislocation

## Abstract

**Background:**

Considerable variability exists in the described clinical and radiographic indications for use, surgical techniques, postoperative management, and risk profile after trochleoplasty for the management of patellofemoral instability (PFI). In areas of clinical uncertainty, a cohesive summary of expert opinion and identification of areas of variation in current practice can be useful in guiding current practice and future research efforts.

**Purpose:**

To assess the current indications for use, surgical techniques, postoperative rehabilitation practices, and observed complication profile for trochleoplasty in the management of PFI among surgeons who perform this procedure.

**Study Design:**

Cross-sectional study.

**Methods:**

A 21-item cross-sectional survey was developed to evaluate trochleoplasty in its current practice among surgeons around the world. The survey was distributed between December 2021 and April 2022 to the orthopaedic surgeon membership of multiple national and international knee, arthroscopy, and sports medicine societies to identify any surgeon with experience performing the trochleoplasty procedure in practice for the management of PFI. Descriptive statistics of survey responses were performed to address study aims, and univariate analyses were performed to compare differences between high- and low-volume trochleoplasty surgeons.

**Results:**

Survey distribution identified 32 orthopaedic surgeons with experience performing the trochleoplasty procedure. Procedural indications were most commonly felt to be met with Dejour classification of B or D on magnetic resonance imaging. Trochleoplasty was felt by most to be appropriate as a primary surgical intervention for PFI. A majority of surgeons utilized a Bereiter (thin-flap) trochleoplasty technique with suture-based fixation and performed concurrent medial patellofemoral ligament reconstruction, but other concomitant procedures varied. Range-of-motion precautions and bracing practices varied among respondents, and arthrofibrosis was the most frequently cited observed complication. High- and low-volume trochleoplasty surgeons differed in their radiographic and age-based indications for the procedure.

**Conclusion:**

Study findings indicated that variation exists in the surgical indicators, technique, and postoperative rehabilitation practices of trochleoplasty surgeons, with specific differences noted between high- and low-volume trochleoplasty surgeons. The results of this survey identified areas of equipoise and treatment variation that should direct future research efforts in the study of the trochleoplasty procedure.

Trochleoplasty^3,6,29^ represents a single tool in the arsenal of the patellofemoral instability (PFI) surgeon and the only surgical technique that directly addresses one of the most commonly observed risk factors in recurrent instability, trochlear dysplasia.^12,13^ Currently, long-term follow-up for this procedure is limited in the literature, with the majority of existing work focused on adult cohorts.^11,17,23^ However, investigations with shorter follow-up performed in the adolescent population have demonstrated promising results with low rates of recurrent instability and good clinical and radiographic outcomes.^4,18^ Overall, rates of complication after trochleoplasty appear on par with other patellar stabilizing techniques, although capture of later stage complications may be limited in some published cohorts.^28^ Some concerns exist due to risks unique to this procedure, such as the potential for contribution to the development of early patellofemoral osteoarthritis.^17,23,28^ However, differentiating degenerative changes occurring due to technical aspects of the procedure from the accumulation of chondral damage due to instability events before surgical intervention may be difficult, and prior work has not clearly made this distinction.^1,16^

Reported indications for trochleoplasty are varied.^19,25^ While most commonly described for use in patients with high-grade dysplasia without patellofemoral arthritis or angular or rotational limb deformity,^6^ others feel it should be used more selectively—such as convex trochlea, where more traditional stabilization techniques may be insufficient—or in the revision setting.^2,8,24,25^ In the realm of pediatric patients, there also remains uncertainty regarding the use of this technique in the skeletally immature. Though traditionally described only for use in patients with closed regional physes,^6^ recent work has highlighted safe utilization of this technique in patients with ≤2 years of growth remaining without evidence of growth disturbance.^18^

Given the known risks of recurrent instability in the setting of trochlear dysplasia,^12,13^ further understanding of appropriate indications for trochleoplasty is important. In addition, understanding variation in technique and postoperative considerations is useful in guiding further research efforts. Thus, the purpose of this study was to summarize current practices and experiences among trochleoplasty surgeons internationally via a cross-sectional survey to guide the development of further study of this intervention. We hypothesized that notable variation would exist among surgeons across all of the studied clinical parameters.

## Methods

### Survey Development and Testing

Literature review and investigator discussion led to the development of a 21-item survey aimed at evaluating trochleoplasty in its current practice among performing surgeons. The survey was constructed and reported in accordance with the published recommendations for survey research as recommended by the Checklist for Reporting Results of Internet E-Surveys.^7^

Survey questions were designed to investigate trochleoplasty indications and use. Surgeon demographic questions included provider practice location, society/organization membership, and training to provide context to responses. Participants were then asked about their experiences regarding trochleoplasty, including indications, surgical technique, and postoperative rehabilitation practices. All survey questions are outlined in the study tables. Preliminary review and testing of the survey was conducted by 3 surgeons (B.A.W., J.A.S. and J.L.P.) and 2 research division personnel (including M.G.B.) for question clarity and appropriateness. The survey was then trialed among members of the Pediatric Research in Sports Medicine Society (PRiSM) Patellofemoral Research Interest Group to garner further feedback before distribution. Adaptive questioning methodology was utilized to increase survey completion rate.^7^ The survey contained a maximum of 40 questions based on responses and was limited to a single webpage. Estimated time to completion was 5 to 7 minutes; however, the duration of the survey was not restricted.

Approval of the study protocol was obtained from our local institutional review board before survey distribution. Approval for survey distribution within each distributing society was additionally obtained when necessary.

### Survey Distribution and Collection

The survey was housed within, and survey responses were collected and managed using, our locally hosted REDCap (Research Electronic Data Capture) tools.^9^ Responses were collected anonymously, and data were stored within the REDCap project, only accessible by study team members.

A convenience sample design was utilized with distribution attempted across numerous overlapping orthopaedic organizational platforms to target surgeons with experience performing the trochleoplasty procedure. Surgeons were invited to participate in the survey via direct email or webpage link distributed according to each group's preferred provider survey methodology. Distribution occurred in a rolling fashion between October 2021 and April 2022 by the following organizations: AGA–Society for Arthroscopy and Joint-Surgery (Gesellschaft für Arthroskopie und Gelenkchirurgie); American Orthopaedic Society for Sports Medicine; Arthroscopy Association of Canada, Chilean Knee Surgeon Chat Group; European Society of Sports Traumatology, Knee Surgery & Arthroscopy; International Society of Arthroscopy, Knee Surgery and Orthopaedic Sports Medicine; and the Latin American Society of Knee Arthroscopy and Sports Medicine. Three sports- and/or patellofemoral-focused societies were contacted for participation, but these organizations elected not to distribute the survey during the requested time frame. Participation was voluntary.

The survey was only accessible via the link provided to the aforementioned groups. A short explanation of the survey including the estimated time to completion and study goals was provided. No financial incentive was given to participants.

### Statistical Analysis

Provider demographics were reviewed before analysis to assess for multiple entries from the same individual. Duplicates or incomplete surveys were excluded. A nonresponse analysis could not be performed due to the methodologic design of the study and protection of anonymity. Descriptive statistics of survey responses were performed to address study aims. Categorical variables were summarized as frequencies and percentages. Participants were free to skip any questions that they did not wish to answer, resulting in some survey questions’ having a lower quantity of responses. Percentages are reported as proportions of all question responses. Survey responses were then compared between low- (<5 procedures per year and/or <25 procedures in career) and high-volume (>5 per year and/or >25 procedures in career) surgeons using univariate testing. All statistical analyses were performed in SPSS Version 27 (IBM Corp).

## Results

### Respondent Demographics

Survey distribution methods yielded an international cohort of 32 respondents with experience performing the trochleoplasty procedure (Table 1). Surgeons had a mean of 14 years (range, 1-39 years) in practice, with varied society memberships (Table 1). Technical procedural education was obtained through a variety of methods, but nearly half of surgeons had learned to perform trochleoplasty on their own outside of traditional training.

**Table 1:** Demographics of Trochleoplasty Survey Respondents (N = 32)

Surgeon Characteristic	Total Sample	Low Volume	High Volume	*P*
	(N = 32)	(n = 21)	(n = 11)	
Year of training completion	2008 (1983-2021)	2008.5 (1983-2021)	2007 (1986-2015)	.71
Region of practice				—
North America	5 (16)	2 (10)	3 (27)	
South America	8 (25)	8 (38)	2 (18)	
Europe	16 (50)	9 (43)	7 (64)	
Asia	2 (6)	2 (10)	0 (0)	
Not reported	1 (3)	0 (0)	1 (9)	
Society membership				—
PRiSM	3 (9)	1 (5)	2 (18)	
IPSG	6 (19)	0 (0)	6 (55)	
AOSSM	7 (22)	3 (14)	4 (36)	
AANA	5 (16)	2 (10)	3 (27)	
ESSKA	21 (66)	13 (62)	8 (73)	
ISAKOS	14 (44)	10 (48)	4 (36)	
None of the above	2 (6)	2 (10)	0 (0)	
Setting(s) where trochleoplasty was learned				—
Residency	8 (25)	5 (24)	3 (27)	
Fellowship (traditional)	8 (25)	5 (24)	3 (27)	
Nontraditional fellowship/observership	8 (25)	5 (24)	3 (27)	
Educational course	5 (16)	3 (14)	2 (18)	
On my own/in practice	14 (44)	11 (52)	3 (27)	
Year first trochleoplasty performed	2015 (1986-2021)	2017 (1986-2021)	2008 (2004-2016)	.08
Trochleoplasty procedures performed annually (estimated)				**<.01**
<5	18 (56)	18 (86)	0 (0)	
5-25	10 (31)	3 (14)	7 (64)	
>25	2 (6)	0 (0)	2 (18)	
Not reported	2 (6)	0 (0)	2 (18)	
Trochleoplasty procedures performed in career (estimated)				**<.01**
<5	9 (28)	9 (43)	0 (0)	
5-25	9 (28)	9 (43)	0 (0)	
26-50	3 (9)	3 (14)	0 (0)	
51-75	1 (3)	0 (0)	1 (9)	
76-100	2 (6)	0 (0)	2 (18)	
>100	8 (25)	0 (0)	8 (73)	

a Data are presented as median (range) or n (%). Dashes indicate comparison not performed. Boldface *P* values indicate a statistically significant difference between high- and low-volume surgeons (*P* < .05). AANA, Arthroscopy Association of North America; ESSKA, European Society for Sports Traumatology, Knee Surgery & Arthroscopy; IPSG, International Patellofemoral Study Group; ISAKOS, International Society of Arthroscopy, Knee Surgery and Orthopaedic Sports Medicine; PRiSM, Pediatric Research in Sports Medicine.

### Surgical Indications for Trochleoplasty

Nearly two-thirds of respondents felt that trochleoplasty was only indicated in skeletally mature individuals. There was no clear consensus regarding the lower age limit among those who considered trochleoplasty in skeletally immature patients. Magnetic resonance imaging–based Dejour classification was the preferred method of radiographic indication for the trochleoplasty procedure, with majority agreement for types B and D trochlea meeting criteria. A substantial number of surgeons (n = 6; 19%) utilized lateral trochlear inclination (LTI) as their primary indication for this procedure, but the threshold LTI measure was highly variable. The majority of surgeons felt trochleoplasty could be considered a primary surgical procedure for PFI. The 7 surgeons performed trochleoplasty for indications other than patellar instability, such as patellofemoral pain without instability. Surgeon responses regarding procedural indications are further detailed in Table 2.

**Table 2:** Surgeon Trochleoplasty Preferences and Experience: Indications^*a*^

Survey Questions	Overall	Low Volume	High Volume	*P*
	(N = 32)	(n = 21)	(n = 11)	
1) Among pediatric patients with patellofemoral instability, what is the *youngest* patient you feel can be appropriately indicated for trochleoplasty?				**.01**
Skeletally mature patients only	20 (63)	17 (81)	3 (27)	
≤2 y until skeletal maturity	6 (19)	2 (10)	4 (36)	
≤3 y until skeletal maturity	0 (0)	0 (0)	0 (0)	
Once the patient has hit puberty	5 (16)	1 (5)	4 (36)	
Prepubescent is OK	0 (0)	0 (0)	0 (0)	
Other^*b*^	1 (3)	1 (5)	0 (0)	
2) With regard to radiographic diagnosis, what is your *preferred* method to diagnose trochlear dysplasia and indicate a trochleoplasty?				**<.01**
Dejour classification on lateral radiograph	6 (19)	6 (29)	0 (0)	
Dejour classification on axial MRI	18 (56)	14 (67)	4 (36)	
LTI angle on axial MRI	6 (19)	0 (0)	6 (55)	
Sulcus angle on axial MRI	1 (3)	1 (5)	0 (0)	
Oswestry-Bristol classification	0 (0)	0 (0)	0 (0)	
Other^*c*^	1 (3)	0 (0)	1 (9)	
None	0 (0)	0 (0)	0 (0)	
3) Which Dejour classification grade(s) do you classify as indicators for trochleoplasty? (Check all that apply)	(n = 24)	(n = 20)	(n = 4)	—
A	0 (0)	0 (0)	0 (0)	
B	18 (75)	15 (75)	3 (75)	
C	8 (33)	6 (30)	2 (50)	
D	23 (96)	19 (95)	4 (100)	
4) What LTI angle do you classify as an indication for trochleoplasty? (free text)	(n = 6)	(n = 0)	(n = 6)	—
<4°	1 (17)	0 (0)	1 (17)	
<5°	2 (33)	0 (0)	2 (33)	
<9°	1 (17)	0 (0)	1 (17)	
<10°	1 (17)	0 (0)	1 (17)	
<12°	1 (17)	0 (0)	1 (17)	
5) What sulcus angle do you classify as an indication for trochleoplasty? (free text)	(n = 1)	(n = 1)	(n = 0)	—
Negative sulcus angle or bump	1 (100)	1 (100)	0 (0)	
6) Can trochleoplasty be indicated as a primary procedure or should it strictly be a revision option?	(n = 32)	(n = 21)	(n = 11)	.12
Can be primary	28 (88)	17 (81)	11 (100)	
Revision only	4 (13)	4 (19)	0 (0)	
7) Do you perform trochleoplasty for any indications other than patellofemoral instability (eg, trochlear dysplasia and patellofemoral pain without instability)?	(n = 32)			**<.01**
Yes^*d*^	7 (22)	0 (0)	7 (64)	
No	23 (72)	21 (100)	2 (18)	
Unanswered	2 (6)	0 (0)	2 (18)	

a Data are presented as n (%). Dashes indicate comparison not performed. Boldface *P* values indicate a statistically significant difference between high- and low-volume surgeons (*P* < .05). LTI, lateral trochlear inclination; MRI, magnetic resonance imaging.

b Other responses: “When they have recurrent dislocation, no matter how many years (you can treat it only with procedures for pediatric patients without bone tunnels).”

c Other responses: “True lateral radiographs and sagittal MRI slice centrally. Next step sulcus angle (or LTI) showing convexity. Do ask for 1 image for deciding when to do a trochleoplasty.”

d Other indications: “Dysplasia and pain”; “Chronic patellofemoral pain and severe trochlear dysplasia with high central height in an anterior-posterior plane on axial view”; “Pain with failed conservative treatment”; “Patellofemoral pain with cartilage defect (usually patellar defects)”; “Cartilage defect patella in combination with non conform trochlea” [sic]; “Pain.”

Surgeons reported utilizing a variety of surgical techniques for trochleoplasty with the majority opting for the Bereiter technique (63%) with suture anchor fixation (91%). All respondents always (72%) or usually (28%) performed medial patellofemoral ligament (MPFL) reconstruction concurrently with the trochleoplasty procedure, while utilization of other bony or soft tissue procedures was highly variable. Some surgeons reported utilizing multiple techniques, as shown by the distribution of responses within “check all that apply” questions. Responses to technical questions are further detailed in Table 3.

**Table 3:** Surgeon Trochleoplasty Preferences and Experience: Techniques and Rehabilitation^*a*^

Survey Question	Overall	Low Volume	High Volume	*P*
	(N = 32)	(n = 21)	(n = 11)	
8) What type(s) of trochleoplasty do you perform? (Check all that apply)				—
Bereiter (groove deepening thin flap)	20 (63)	11 (52)	9 (82)	
Dejour (groove deepening thick flap)	14 (44)	12 (57)	2 (18)	
Arthroscopic	3 (9)	1 (5)	2 (18)	
Goutallier (recession wedge)	2 (6)	0	2 (18)	
Albee (lateral facet elevation)	0	0	0	
Other	0	0	0	
9) What type(s) of fixation do you use for your trochleoplasty? (Check all that apply)				—
Suture anchors/absorbable suture	29 (91)	18 (86)	11 (100)	
Screws	6 (19)	6 (29)	0 (0)	
Staples	0 (0)	0 (0)	0 (0)	
Other^*b*^	1 (3)	0 (0)	1 (9)	
None/no fixation	0 (0)	0 (0)	0 (0)	
10) Do you perform MPFL reconstruction at the time of trochleoplasty?				.12
Always (100% of the time)	23 (72)	17 (81)	6 (55)	
Usually (roughly ≥80% of the time)	9 (28)	4 (19)	5 (45)	
11) Do you perform medial retinacular plication at the time of trochleoplasty?				.82
Always (100% of the time)	1 (3)	1 (5)	0 (0)	
Usually (roughly ≥80% of the time)	1 (3)	1 (5)	0 (0)	
Often (>50% of the time)	2 (6)	1 (5)	1 (9)	
Sometimes (<50% of the time)	2 (6)	2 (10)	0 (0)	
Rarely (roughly 20% of the time)	9 (28)	6 (29)	3 (27)	
Never (0% of the time)	15 (47)	10 (48)	5 (45)	
Unanswered	2 (6)	0 (0)	0 (0)	
12) Do you perform a lateral retinacular release or lengthening at the time of trochleoplasty?				.57
Always (100% of the time)	7 (22)	4 (19)	3 (27)	
Usually (roughly ≥80% of the time)	4 (13)	2 (10)	2 (18)	
Often (>50% of the time)	4 (13)	2 (10)	2 (18)	
Sometimes (<50% of the time)	6 (19)	5 (24)	1 (9)	
Rarely (roughly 20% of the time)	6 (19)	5 (24)	1 (9)	
Never (0% of the time)	2 (6)	2 (10)	0 (0)	
Unanswered	3 (9)	1 (5)	2 (18)	
13) If, in the setting of high-grade/severe trochlear dysplasia that meets your indication for a trochleoplasty, the TT-TG is also elevated to a level that is considered a surgical indication for a TTO, would you perform both a trochleoplasty and a TTO?				.28
No, the trochleoplasty will address the TT-TG	8 (25)	4 (19)	4 (36)	
Yes, always	3 (9)	2 (10)	1 (9)	
Yes, but only if the TT-TG is >20-25 mm	13 (41)	11 (52)	2 (18)	
Other (free text)^*c*^	8 (25)	4 (19)	4 (36)	

a Data are presented as n (%). Dashes indicate comparison not performed. MPFL, medial patellofemoral ligament; TT-TG, tibial tubercle–trochlear groove distance; TTO, tibial tubercle osteotomy; TTPCL, tibial tubercle–posterior cruciate ligament.

b Other response: “Smart nail.”

c Other responses: “In cases of very high TT-TG, I consider either TTO or rotational tibial osteotomy”; “Try to correct TT-TG with trochleoplasty lateralizing the trochlea. If it is still not possible to normalize the TT-TG then I add TTO”; “Check the TTPCL and decide if TTO is necessary”; “Depends on TTPCL distance”; “Individually”; “Only in very severe situations where TT-TG is not corrected by trochleoplasty”; “Depends on the TT-TG distance and the correction achieved with trochleoplasty”; “Yes, but not always.”

### Postoperative Precautions, Imaging, and Complications

Postoperative practices varied considerably among respondents (Table 4). The majority allowed for full (44%) or partial (41%) weightbearing with some degree of immediate postoperative motion permitted. There was equipoise among respondents regarding the necessity of bracing. Postoperative imaging practices were also highly variable with the only majority response among surgeons being radiographs obtained at 6 weeks postoperatively. The majority (72%) did not obtain magnetic resonance imaging routinely in the postoperative setting. Arthrofibrosis, chondrolysis, or progressive patellofemoral arthritis were the most common complications observed by surgeons. No surgeon reported the identification of clinically significant growth disturbances observed when utilized in skeletally immature patients when growth remaining was ≤2 years.

**Table 4:** Surgeon Trochleoplasty Preferences and Experience: Postoperative Follow-up and Complications^*a*^

Survey Question	Overall	Low Volume	High Volume	*P*
	(N = 32)	(n = 21)	(n = 11)	
14) What weightbearing restrictions do you employ during the first 4-6 weeks after surgery?				.40
Weightbearing as tolerated	14 (44)	7 (33)	7 (64)	
Partial weightbearing	13 (41)	10 (48)	3 (27)	
Toe-touch weightbearing	2 (6)	2 (10)	0	
Nonweightbearing	1 (3)	1 (5)	0	
Other^*b*^	2 (6)	1 (5)	1 (9)	
15) What range of motion restrictions do you employ during the first 4-6 weeks after surgery?				.05
No motion restrictions	13 (41)	6 (29)	7 (64)	
Range of motion between 0° and 90°	6 (19)	4 (19)	2 (18)	
Some sort of progressive increase (ie, 0°-30° for 2 weeks, 0°-60° for 2 additional weeks, etc)	12 (37.5)	11 (52)	1 (9)	
Other^*c*^	1 (3)	0	1 (9)	
16) Do you utilize a hinged knee brace in the immediate postoperative period?				.17
Yes	14 (44)	11 (52)	3 (27)	
No	18 (56)	10 (48)	8 (73)	
17) Do you routinely obtain radiographs at any time point during the postoperative period following a trochleoplasty?				.37
Yes	26 (81)	18 (86)	8 (73)	
No	6 (19)	3 (14)	3 (27)	
18) Please indicate the time points at which you obtain radiographs. (Check all that apply)	(n = 26)	(n = 18)	(n = 8)	—
6 weeks postop	20 (77)	13 (72)	7 (88)	
3 months postop	13 (50)	10 (56)	3 (38)	
6 months postop	9 (35)	8 (44)	1 (13)	
1 y postop	13 (50)	11 (61)	2 (25)	
Other^*d*^	2 (8)	2 (11)	0	
19) Do you routinely obtain MRI at any time point during the postoperative period following a trochleoplasty?				.94
Yes	9 (28)	6 (29)	3 (27)	
No	23 (72)	15 (71)	8 (73)	
20) Please indicate the time points at which you obtain an MRI. (Check all that apply)	(n = 9)	(n = 6)	(n = 3)	—
6 weeks postop	0	0	0	
3 months postop	1 (11)	0	1 (33)	
6 months postop	4 (44)	4 (67)	0	
1 y postop	5 (56)	3 (50)	2 (67)	
Other	0	0	0	
21) Have you observed any of the following in your practice in patients undergoing trochleoplasty for patellofemoral instability? (Check all that apply)				—
Arthrofibrosis requiring manipulation/revision surgery	19 (59)	12 (57)	7 (64)	
Osteochondral flap failure/nonunion	2 (6)	1 (5)	1 (9)	
Chondrolysis/progressive patellofemoral osteoarthritis	5 (16)	3 (14)	2 (18)	
Implant-related complication requiring return to OR	3 (9)	2 (10)	1 (9)	
Clinically significant growth disturbance (if used in a skeletally immature patient)	0	0	0	
Recurrent instability/redislocation	2 (6)	1 (5)	1 (9)	
Other^*e*^	4 (13)	1 (5)	3 (27)	
Unanswered/none of the above	7 (22)	5 (24)	2 (18)	

a Data are presented as n (%). Dashes indicate comparison not performed. DVT, deep vein thrombosis; MRI, magnetic resonance imaging; OR, operating room; postop, postoperative; WBAT, weightbearing as tolerated.

b Other responses: “WBAT with braced locked in mild flexion (10°-15°)”; “Trochleo + osteotomy = always weightbearing.”

c Other response: “No full extension for 2 weeks, 10° restriction.”

d Other responses: “Immediately postop”; “2 days postop.”

e Other responses: “DVT and pain”; “Infection”; “Focal chondral necrosis from overtensioning sutures” (2 responses).

### Volume-Based Surgeon Comparisons

Survey responses comparing high-volume and low-volume surgeons (Tables 1-4) identified no significant differences in demographics, including years in practice, region of practice, or society memberships. High-volume surgeons demonstrated a lower age threshold for indicating trochleoplasty in pediatric patients with PFI, with a significant majority considering the procedure in patients before skeletal maturity (high vs low volume: 73% vs 19%; *P* = .01). High-volume surgeons also showed a preference for utilizing LTI as a radiographic indicator for trochleoplasty, while none of the low-volume surgeons preferred this measure (high vs low volume: 55% vs 0%; *P* < .01). Finally, a majority of high-volume surgeons considered trochleoplasty for indications other than PFI (high vs low volume: 64% vs 0%; *P* < .01). No other significant differences were noted between surgeon groups with regard to surgical technique and postoperative practices.

## Discussion

The presented survey describes the current practices of a large cohort of surgeons who perform an uncommon surgical procedure. It provides a comprehensive summary of current surgeon utilization and practices regarding trochleoplasty across a diverse international cohort of surgeons with varied experience with the procedure. In areas of limited evidence, expert opinion can provide provisional guidelines and set a foundation for practice. Additionally, areas lacking consensus demonstrate clinical equipoise, which can be useful in guiding future research efforts. Survey results indicated that variation exists in the radiographic and age-based indications for trochleoplasty and the necessity of performing concurrent bony and soft tissue procedures. Surgical technique preferences showed slightly more agreement. Postoperative rehabilitation precautions and radiographic follow-up were highly variable. Arthrofibrosis was a commonly encountered postoperative complication, highlighting the need for early mobilization. Growth disturbance was not observed in the surveyed cohort.

Trochlear morphological indications for trochleoplasty reported in the literature are variable.^19,25^ While some advocate for use in settings of both flat or convex trochlear morphology,^6^ others feel it should be used more selectively for only convex trochlea or for patients who have previously failed other surgical interventions.^2,8,24,25^ Our study identified that the current practices of trochleoplasty are varied and with limited consensus regarding these indications. We found that both the Dejour classification and LTI were predominantly used for appropriately indicating this procedure despite concerns existing regarding the reliability of the Dejour classification.^26,27^ Surgeon trochleoplasty volume appeared to be associated with surgeon utilization of these radiographic assessments. For lower-volume surgeons who favored the Dejour classification, the vast majority of respondents felt flat (Dejour B) and convex (Dejour D) trochlea, but consideration for Dejour C and even Dejour A morphology was still considered by some. Among higher-volume surgeons who favored LTI, a strict quantitative cutoff was not identified. Efforts are therefore necessary to both determine the best assessment for characterizing trochlear morphology and improve consensus regarding appropriate use criteria for the trochleoplasty procedure. Last, while trochlear bump height^21,22^ was not included on the survey as a unique indication for trochleoplasty, it was endorsed as a free response and its role may require further exploration in future directions of this research.

Beyond the trochleoplasty procedure itself and its indications, our survey additionally identified variability among surgeons as it relates to the necessity of concurrent procedures as well as postoperative rehabilitation guidelines. While the vast majority of surgeons perform MPFL reconstruction at the time of trochleoplasty, there is substantial variation in performing concurrent procedures including tibial tubercle osteotomy or lateral soft tissue lengthening or release. Postoperatively, rehabilitation practices after trochleoplasty among responding surgeons also had noted differences, particularly with relation to weightbearing limitations and motion progression. This is unsurprising given the variation of rehabilitation practices that has been observed within the realm of MPFL reconstruction alone.^10,14,15^ Future work should seek to evaluate the necessity of postoperative motion and weightbearing restrictions and their impact on the risk of arthrofibrosis, given this is a frequently cited concern both among responding surgeons and within the existing literature.^11^

Complications after trochleoplasty are varied, but prior work has demonstrated their frequency to be similar to other patellar stabilizing procedures.^5,11,28^ Paralleling the existing literature, the most frequently cited complication among our surveyed cohort was arthrofibrosis which was observed by the majority of surgeons. The second most common complication cited by respondents was chondrolysis or patellofemoral arthritis (16%). Surgeons did not indicate the method by which this was diagnosed or the duration their patients were followed and thus the prevalence of this may be underappreciated with this finding. Nevertheless, a cautious approach is recommended and appropriate counseling should be performed when trochleoplasty is considered in younger patients. Recurrent instability or dislocation rates appeared consistently <10% in prior studies^5,11,28^ despite this procedure's being performed largely in patients with high-grade trochlear dysplasia. Within our surveyed cohort, recurrent instability or dislocation was also infrequently cited, even among high-volume trochleoplasty surgeons. While this result may be affected by recall bias, it does provide support for the efficacy of the procedure with regard to enhancing stability in a high-risk population. Interestingly, flap nonunion failure or nonunion was reported by a few survey respondents despite not being well described in the literature. Possible reasons for failure of the flap to heal include too thin of a flap, excessive removal of subchondral bone, failure of fixation, or avascular necrosis. This may warrant further exploration to improve our understanding of this complication.

Skeletal immaturity is widely considered a contraindication for trochleoplasty due to the proximity of the distal femoral physis to the trochlea^20^ and concerns of anterior physeal bar formation at the osteotomy site.^6^ While recent work among adolescents nearing skeletal maturity (within ~2 years) demonstrated no growth disturbance using a Bereiter technique, the proximity of this cohort to maturity may have made premature physeal arrest or any resultant deformity difficult to detect.^18^ Our survey indicated variable opinion regarding trochleoplasty use as it relates to skeletal age with higher-volume surgeons having a greater willingness to consider this intervention in skeletally immature individuals. It is prudent for the surgeon to be able to estimate remaining growth, as intervention >2 years before skeletal maturity or in prepubertal period was not endorsed by any surgeon. Of note, none of the surveyed surgeons had identified growth disturbance as a complication from the trochleoplasty procedure, but it is unclear what methods are being used and how closely these patients are being monitored for this complication. Given the high rate of trochlear dysplasia and increased risk of recurrent instability among pediatric patients, future research should seek to determine the impact of trochleoplasty techniques on the distal femoral physis to further clarify appropriate indications for this technique.

### Limitations

The presented study is not without limitations, most of which are related to the convenience sample study design utilized. This approach was selected due to the assumption that the intended audience (surgeons with trochleoplasty experience) represented a small percentage of surgeons within the many surveyed organizations. Many of these individuals were also known to be members of multiple related organizations, and thus determination of a true denominator of distribution was not feasible. Additionally, while near-simultaneous distribution by 10 organizations was attempted to maximize responses, differential organizational approval timelines and distribution methods resulted in unintended variation in survey release timing and access. Some organizations also elected not to distribute the survey at all, which was out of the control of the study investigators. The study design also carried inherent sampling bias and limits any inferences regarding survey nonresponders. Further, there was selection bias of the presented results toward English-speaking surgeons given that the survey was only distributed in the English language. Despite these limitations, the respondents demonstrated broad international representation across multiple continents. Finally, there were limitations with regard to the survey content and associated bias of its design. The survey did not capture nonradiographic aspects of decision making as it relates to physical examination findings such as ligamentous laxity. The survey also did not fully capture the practice makeup (eg, volume of pediatric versus adult patients) of the responding surgeon and relied instead on region, training, and volume and years of experience. Additionally, all responses were subject to recall bias, particularly with regard to operative volume and postoperative complications. Nevertheless, we feel this information still serves a valuable purpose of making surgeons aware of complications that have been observed by others. Also, practices endorsed by surgeons in this survey cannot be considered benchmarks or guidelines until they are further validated in a scientific manner.

## Conclusion

This study provided a summary of current provider opinion and experience regarding the use of trochleoplasty in the management of PFI. Findings from this study indicated that across a diverse, international cohort of trochleoplasty surgeons, considerable variation exists in surgical indications, technique, and postoperative management. Survey results provide a template for defining appropriate practices and may prove a useful reference for surgeons interested in adopting this procedure in their surgical armamentarium. Numerous areas of clinical equipoise were identified, laying the groundwork for directing future research efforts in the area of trochleoplasty.
